# Novel Insights Into the Interaction Between the Autonomic Nervous System and Inflammation on Coronary Physiology: A Quantitative Flow Ratio Study

**DOI:** 10.3389/fcvm.2021.700943

**Published:** 2021-07-27

**Authors:** Jun Wang, Wei Liu, Huaqiang Chen, Chengzhe Liu, Meng Wang, Hu Chen, Huixin Zhou, Zhihao Liu, Song Zhang, Zhongyang Yu, Shoupeng Duan, Qiang Deng, Ji Sun, Hong Jiang, Lilei Yu

**Affiliations:** Department of Cardiology, Renmin Hospital of Wuhan University, Cardiac Autonomic Nervous System Research Centre of Wuhan University, Cardiovascular Research Institute, Wuhan University, Hubei Key Laboratory of Cardiology, Wuhan, China

**Keywords:** unstable angina pectoris, quantitative flow ratio, heart rate variability, autonomic nerve, inflammation

## Abstract

**Background:** Heart rate variability (HRV) was proposed as a noninvasive biomarker to stratify the risk of cardiovascular disease. However, it remains to be determined if HRV can be used as a surrogate for coronary artery physiology as analyzed by quantitative flow ratio (QFR) in patients with new-onset unstable angina pectoris (UAP).

**Methods:** A total of 129 consecutive patients with new-onset UAP who underwent 24-h long-range 12-channel electrocardiography from June 2020 to December 2020 were included in this study. HRV, coronary angiography, and QFR information was retrieved from patient medical records, the severity of coronary lesions was evaluated using the Gensini score (GS), and total atherosclerotic burden was assessed using the three-vessel contrast QFR (3V-cQFR) calculated as the sum of cQFR in three vessels.

**Results:** Multivariate logistic analysis showed that low-frequency power (LF) and high-sensitivity C-reactive protein (hs-CRP) were directly correlated with functional ischemia of target vessel, which were inversely correlated with total atherosclerotic burden as assessed by 3V-cQFR. Moreover, incorporation of the increase in LF into the existing model that uses clinical risk factors, GS, and hs-CRP significantly increased the discriminatory ability for evaluating coronary artery physiology of target vessel.

**Conclusions:** LF and hs-CRP are independently associated with functional ischemia in patients with new-onset UAP. The relative increase of LF and hs-CRP could add value to the use of classical cardiovascular risk factors to predict the functional severity of coronary artery stenosis. Our results suggest a potential association between the autonomic nervous system, inflammation, and coronary artery physiology.

## Introduction

Traditional cardiovascular risk factors, such as age, family history of premature heart disease, diabetes, cigarette smoking, hypertension, and dyslipidemia, have been demonstrated to be linked to the incidence of acute cardiovascular events and the increased risks of morbidity, mortality, and disability in patients with acute coronary syndrome (ACS) (1). However, acute coronary events can occur in healthy individuals without the above-mentioned traditional cardiovascular risk factors, suggesting the presence of unrecognized risk factors (1, 2). Pathologically, the incidence of acute coronary events has been associated with functionally significant coronary artery stenosis and total physiological atherosclerotic burden (3–6). Therefore, identifying hidden risk factors correlated with functional severity of coronary artery stenosis and total atherosclerotic burden would be important for predicting acute coronary events in patients with ACS, particularly in those with unstable angina pectoris (UAP).

Strikingly, the heart rate variability (HRV) measurement, a simple and non-invasive technique that provides assessment of autonomic modulation of cardiac regulation, can independently predict the mortality of patients with no known history of cardiovascular disease (7). HRV has been documented in patients at higher risk for ACS, such as those with diabetes, hypertension, and high low-density lipoprotein cholesterol (8–11). Moreover, low-frequency power (LF) is highly predictive for coronary artery disease (CAD) regardless of the traditional cardiovascular risk factors, and it is a potential clinical risk stratification tool in patients with sinus rhythm (12). Clinical studies have also demonstrated an inverse correlation between HRV and chronic low-grade systemic inflammation in patients with stable CAD (13). Accumulating evidence demonstrates that the autonomous nervous system (ANS) plays an important role in the regulation of systemic inflammation, and an ANS imbalance may contribute to the increased risk of acute cardiovascular events via promoting inflammation and endothelium damage (14–16). However, whether the balance of the ANS evaluated by HRV and inflammation is independently associated with functionally significant coronary artery stenosis in patients with ACS remains to be determined.

Compared to the traditional coronary angiogram, quantitative flow ratio (QFR) has become a popular tool to more accurately evaluate the functional severity of coronary artery stenosis based on three-dimensional quantitative angiography and fluid dynamics algorithms (17). The QFR measurement does not require the use of pressure-wires, hyperemia induction, or reconstruction of all side branches, compared to fractional flow reserve (FFR) (18). Moreover, in terms of diagnosing functional coronary artery stenosis, QFR is highly consistent with FFR, the gold standard for evaluating functional stenosis of coronary arteries (18). Although the association between HRV, inflammation, and the severity of coronary angiographic stenosis has been confirmed (12, 19), there remains no literature providing any evidence that HRV and inflammation are correlated with the severity of functional coronary artery stenosis as detected by QFR. The aim of the current study was to systematically evaluate the potential association between HRV, inflammation, and coronary physiology in new-onset UAP patients.

## Methods

### Patient Population

A total of 129 consecutive patients with a first diagnosis of new-onset UAP and no known history of CAD who underwent coronary angiography and QFR measurement at the Department of Cardiology of Renmin Hospital of Wuhan University from June 2020 to December 2020 were included in this retrospective study. Diagnosis of UAP was made in accordance with previously established guidelines (20). Patients were excluded if they had the following conditions: previous paroxysmal or atrial fibrillation, implantation of pacemaker, other arrhythmias (e.g., frequent premature beat, bradycardia, sick sinus syndrome, atrioventricular block, broad bundle branch blocks, or ventricular arrhythmias), depressive disorder, hyperthyroidism, excessive drinking, inflammatory disease, malignant tumors, acute or chronic infection, any systemic acute disease, taking any medications affecting HRV, history of CAD, variable angina, aggravated effort type angina pectoris, valvular heart disease, and congenital heart disease. For QFR analysis, patients with either a prolonged occluded coronary bypass graft, myocardial bridge, ostial lesions, severe vessel overlap or tortuosity at the stenotic segments, side branch lesions, or poor angiographic image quality where QFR measurement could not be constructed were excluded. Because this was a retrospective observational study, the Renmin Hospital of Wuhan University Ethics Committee granted an exemption from requiring ethics approval and informed consent from eligible patients was waived.

### Blood Tests

Venous blood samples were obtained upon admission from each patient before coronary angiography and analyzed for lipid profile, inflammation biomarkers (high-sensitivity C-reactive protein (hs-CRP), interleukin 2 (IL-2), IL-4, IL-6, IL-10, and fibrinogen), and other blood biochemical routine tests at the Department of Clinical Laboratory in Renmin Hospital of Wuhan University.

### Holter Monitoring and HRV Analysis

For all participants, a 24-h long-range 12-channel electrocardiography recording was applied for HRV analysis before coronary angiography. The 24-h mean heart rate and variables of HRV were automatically analyzed using commercially available software (H-Scribe Analysis System, Mortara Instrument, Inc., Milwaukee, WI, USA). The specific signal processing steps for computation of the HRV parameters were the same as described previously (21–23). The frequency domain analysis method was used to analyze the 5-minute short-term with relatively stable RR interval by Fourier transform, where the power spectrum graph was created using frequency (Hz) as the abscissa coordinate and power spectral density as the ordinate coordinate. The average 5-minute short-term HRV analysis was performed in our study. The spectrum of the short-range record is divided into three frequency bands, where VLF, LF, and HF are the integrated power spectral density (PSD) values in different frequency bands. (1) TP (total power): total frequency, frequency spectra ≤ 0.4 Hz, representing total variations of normal-to-normal R-R intervals (NN intervals), (2) HF (high frequency power): high frequency, frequency spectra 0.15–0.4 Hz, (3) LF (low frequency power): low frequency, frequency spectra 0.04–0.15 Hz, (4) very low-frequency (VLF): very low frequency, frequency spectra 0.003–0.04 Hz. Absolute spectral power values are expressed in squared milliseconds (ms^2^). Normalized LF power was calculated as LFn = 100^*^LF/(total power-VLF), and normalized HF power was calculated as HFn = 100^*^HF/(total power-VLF). HF and HFn generally reveal cardiac parasympathetic nerve modulation, while LF and LFn are possibly correlated to sympathetic modulation or to autonomic balance. Accordingly, the LF/HF ratio is an indicator of the balance of the cardiac autonomic nervous system (24).

### Coronary Angiography and Gensini Score (GS)

All participants were scheduled to undergo coronary artery angiography. The severity of stenosis of the coronary artery was independently evaluated by two investigators, and consensus was required with a third investigator if any disagreements arose. Target vessel was defined as the vessel with the most severe lesion. The GS, which integrates the extent of luminal narrowing and the geographic importance of the lesion, was calculated to reflect the severity of coronary artery lesions (25).

### QFR Computation

**Figure 1:**
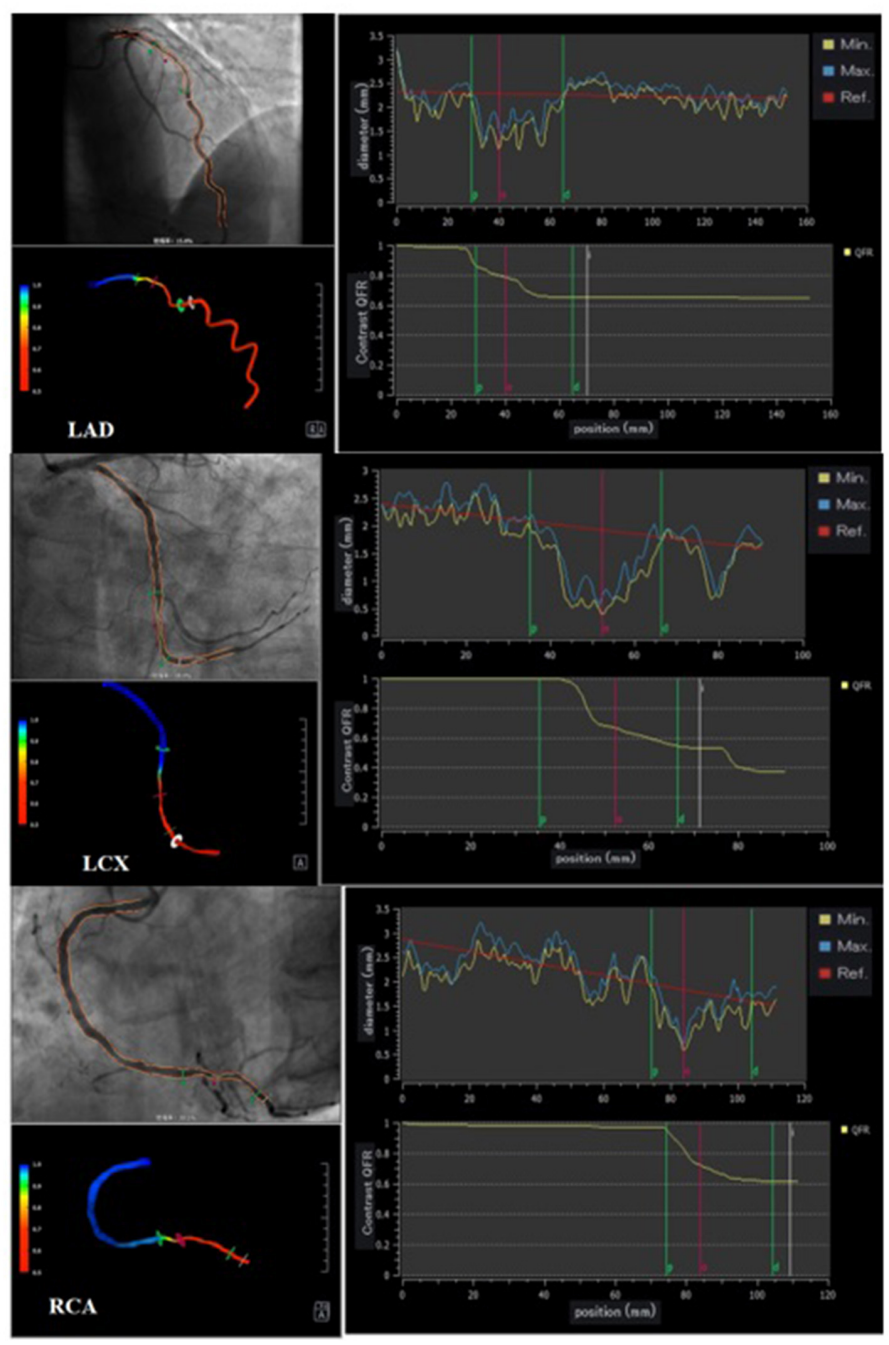
Representative cQFR analysis. Three representative cases undergoing quantitative flow ratio (QFR) measurement. Vessel contours were delineated from two different views of coronary angiograms acquired with projection angles at a minimum of 25° apart (left upper panel). During QFR computation, quantitative image analysis of 3D reconstruction provides further insights into the quantitative anatomical parameters of each vessel (left lower panel); contrast-flow QFR (cQFR) is shown on the right panels.

Three vessels in each patient were analyzed using offline QFR and computed using the AngioPlus system (Pulse Medical Imaging Technology, Shanghai, China). The two selected views with a projection angle of a minimum of 25° apart in the angiographic image were transferred to the QFR system through the local network. A modified TIMI frame count method was used for QFR computation. Recent evidence indicates that in the absence of drug-induced hyperemia, the cQRF obtained through modeled hyperemic flow velocity of the coronary artery shows good consistency with the gold standard FFR (17, 26). In our study, cQFR that was obtained from routine angiographic images was used to assess the severity of the functional coronary lesion. Three-vessel cQFR (3V-cQFR) calculated as the sum of cQFR in three vessels has been shown to reflect the total atherosclerotic burden, a variable that can be used to identify “vulnerable” patients (6). Representative examples of the quantitative image analysis of three-dimensional reconstruction and QFR computation are demonstrated in Figure 1.

### Statistical Analysis

Patients were grouped according to the tertiles of the GS. All statistical analyses were performed using SPSS23 software. Comparisons between two groups were performed using an independent sample *t*-test or the Mann-Whitney U test. Categorical data are presented as counts (proportions) and were compared using the χ^2^ test or Fisher's exact test. Multiple stepwise logistics regression analysis was performed to identify factors associated with the GS and target vessel with QFR ≤ 0.8. Linear regression analysis was used to identify factors associated with the 3V-cQFR. ROC curve analysis was used to show the predictability of target vessel with QFR ≤ 0.8 using GS, hs-CRP, and LF. We compared whether adding GS, hs-CRP, and LF to the traditional cardiovascular risk factors would improve the discriminant and reclassification ability of the models. Continuous data are presented as mean ± standard deviation (SD) or median (interquartile range), and categorical data are presented as numbers and percentages. A two-sided *P* < 0.05 was considered statistically significant.

## Results

### Patient Characteristics According to cQFR of Target Vessel

**Figure 2:**
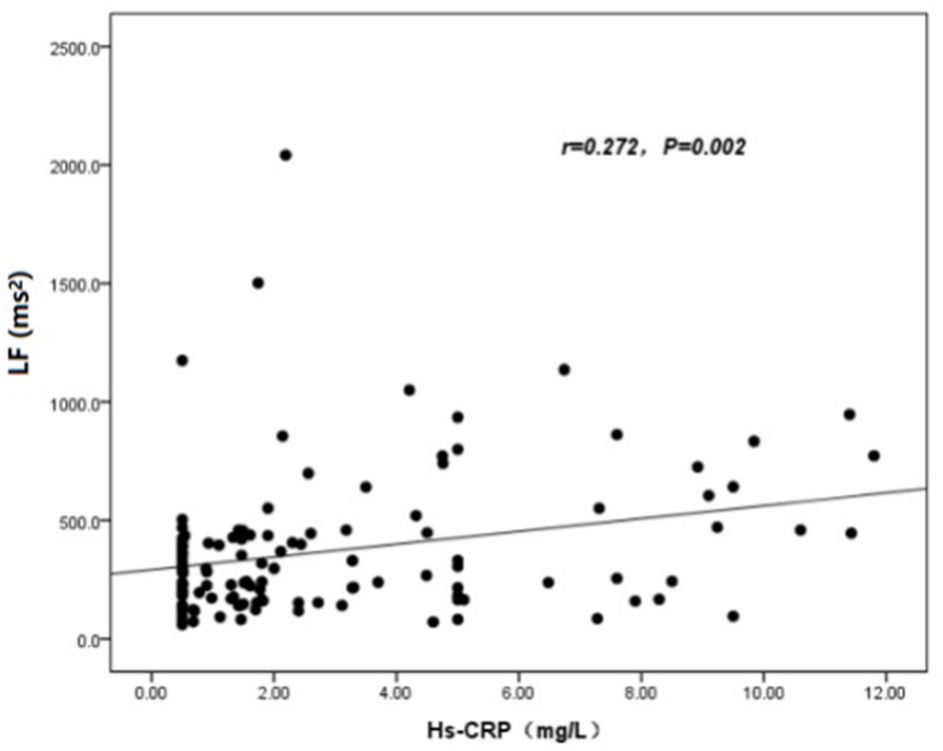
Association analysis between hs-CRP and LF.

The baseline features of patients based on cQFR of target vessel (cQFR of target vessel ≤ 0.8, *n* = 77 and cQFR of target vessel > 0.8, *n* = 52) are presented in Table 1. Those with cQFR ≤ 0.8 of target vessel were mostly male, with lower high-density lipoprotein cholesterol (HDL-C), and higher hs-CRP, GS, LFn, and LF compared to those with cQFR > 0.8 of target vessel. There were no correlations between other indicators and cQFR of target vessel ≤ 0.8 (all *P* > 0.05). LF was positively correlated with hs-CRP (*r* = 0.272, *P* < 0.01; Figure 2). Multivariable stepwise logistic regression analysis showed that both hs-CRP and LF were independent predictors of functional severity of coronary stenosis of target vessel (all *P* < 0.05; Table 2).

**Table 1:** Patient characteristics according to cQFR of target vessel.

	**cQFR of target vessel > 0.8** **(***n*** = 52)**	**cQFRof target vessel ≤ 0.8** **(***n*** = 77)**	**t/Z/χ^2^**	***P***
Male (%)	**27 (51.9)**	**57 (74.0)**	**6.676**	**0.010**
Age (years)	63.73 ± 9.31	61.56 ± 9.54	1.281	0.203
Hypertension (%)	35 (67.3)	53 (68.8)	0.033	0.855
Diabetes mellitus (%)	13 (25.0)	26 (33.8)	1.131	0.288
Current smoking (%)	18 (34.6)	38 (49.4)	2.743	0.098
Family history of CAD (%)	13 (25.0)	21 (27.3)	0.083	0.774
BMI (kg/m^2^)	3 (5.8)	9 (11.7)	0.683	0.409
TG (mmol/l)	25.54 ± 3.56	25.92 ± 3.95	0.563	0.574
TC (mmol/l)	1.48 (0.93, 2.08)	1.48 (1.20, 2.13)	0.854	0.393
**HDL-c (mmol/l)**	**1.21 ± 0.37**	**1.04 ± 0.25**	**3.049**	**0.003**
LDL-c (mmol/l)	2.42 ± 0.96	2.65 ± 1.25	1.112	0.268
Lp(a) (g/L)	127.00 (63.00, 210.00)	150.00 (85.00, 343.50)	1.618	0.106
hs-CRP (mg/L)	**0.94 (0.50, 2.40)**	**3.18 (1.48, 7.60)**	**5.015**	**<0.001**
IL-2 (pg/ml)	3.24 (2.81, 3.89)	2.88 (2.60, 3.60)	1.208	0.227
IL-4 (pg/ml)	2.21 (1.86, 2.53)	1.95 (1.78, 2.15)	1.888	0.059
IL-6 (pg/ml)	6.52 (4.67, 11.50)	7.10 (5.65, 11.94)	1.085	0.278
IL-10 (pg/ml)	4.03 (3.60, 5.20)	3.75 (3.39, 4.18)	1.063	0.288
Fibrinogen (g/L)	2.61 ± 0.99	2.95 ± 1.32	1.581	0.116
Average heart rate (beats/min)	63.73 ± 9.31	61.56 ± 9.54	1.694	0.093
Total power (ms^2^)	1581.95 (1059.85, 2400.23)	1857.70 (1290.40, 2702.10)	1.647	0.100
Normalized LF power norm (nu)	62.94 (47.24, 69.39)	66.15 (58.35, 74.42)	2.290	**0.022**
Normalized HF power norm (nu)	27.84 (14.84, 36.38)	28.19 (20.03, 37.77)	0.538	0.591
LF (ms^2^)	**209.95 (142.83, 347.68)**	**396.40 (227.05, 641.30)**	**4.153**	**<0.001**
HF (ms^2^)	102.30 (41.83, 194.93)	135.90 (73.75, 230.95)	1.642	0.101
LF/HF	2.14 (1.31, 3.47)	2.65 (1.65, 3.87)	1.575	0.115
**Vessel analysis**				
Gensini score	**13.75 (8.13, 40.88)**	**46.00 (21.25, 83.25)**	**4.214**	**<0.001**
Gensini score group			**27.514**	**<0.001**
1^st^ tertile	**31 (59.6)**	**12 (15.6)**		
2^nd^ tertile	**9 (17.3)**	**34 (44.2)**		
3^rd^ tertile	**12 (23.1)**	**31 (40.3)**		
Target vascular location			4.249	0.120
LAD	31 (59.6)	40 (51.9)		
LCX	14 (26.9)	15 (19.5)		
RCA	7 (13.5)	22 (28.6)		

*The meaning of the bold values are a two-sided P <0.05 was considered statistically significant*.

**Table 2:** Independent predictors of cQFR of target vessel ≤0.8.

**Independent variables**	**B**	**SE**	**WALS**	***P***	**OR**	**95%**	
						**Lower limit**	**Upper limit**
Female	0.297	0.519	0.328	0.567	1.346	0.487	3.722
HDL-c	−1.581	0.869	3.308	0.069	0.206	0.037	1.131
hs-CRP	**0.196**	**0.086**	**5.141**	**0.023**	**1.216**	**1.027**	**1.441**
LF	**0.004**	**0.001**	**8.043**	**0.005**	**1.004**	**1.001**	**1.006**

*The meaning of the bold values are a two-sided P <0.05 was considered statistically significant*.

### Patient Characteristics According to GS

The baseline features of patients based on the tertiles of GS were 1^st^ tertile GS < 16.5, *n* = 43; 2^nd^ tertile GS: 16.5–52.5, *n* = 43; and 3^rd^ tertile GS > 52.5, *n* = 43 (Table 3). The percentages of being male, current smoking, HDL-C, hs-CRP, IL-6, 3V-cQFR, and cQFR of target vessel were significantly different among the groups based on GS tertiles (all *P* < 0.05). However, there was no correlation between other indicators and the severity of coronary lesions (all *P* > 0.05). Multivariable stepwise logistic regression analysis showed that both HDL-C and hs-CRP were independent predictors of GS (all *P* < 0.05; Table 4).

**Table 3:** Patient characteristics according to Gensini Score.

	**1^st^ tertile <16.5** **(***n*** = 43)**	**2^nd^ tertile 16.5–52.5** **(***n*** = 43)**	**3^rd^ tertile > 52.5** **(***n*** = 43)**	**F/Z/χ^2^**	***P***
Male (%)	**22 (51.2)**	**29 (67.4)**	**33 (76.7)**	**6.348**	**0.042**
Age (years)	60.09 ± 8.64	64.74 ± 9.09	62.47 ± 10.25	2.660	0.074
Hypertension (%)	33 (76.7)	30 (69.8)	25 (58.1)	3.504	0.173
Diabetes mellitus (%)	11 (25.6)	15 (34.9)	13 (30.2)	0.882	0.643
Current smoking (%)	**12 (27.9)**	**20 (46.5)**	**24 (55.8)**	**7.068**	**0.029**
Family history of CAD (%)	5 (11.6)	3 (7.0)	4 (9.3)	0.474	0.624
BMI (kg/m^2^)	26.19 ± 3.61	25.71 ± 3.73	25.39 ± 4.05	0.345	0.710
TG (mmol/l)	1.52 (0.93, 2.32)	1.42 (1.17, 1.88)	1.49 (1.25, 2.11)	0.390	0.823
TC (mmol/l)	4.48 ± 1.35	4.12 ± 1.23	4.33 ± 1.39	0.812	0.446
HDL-c (mmol/l)	**1.22 ± 0.40**	**1.09 ± 0.26**	**1.02 ± 0.22**	**4.801**	**0.010**
LDL-c (mmol/l)	2.57 ± 1.18	2.36 ± 0.94	2.75 ± 1.27	1.243	0.292
Lp(a) (g/L)	121.50 (61.50, 287.75)	165.00 (89.00, 332.00)	139.00 (81.00, 263.00)	2.175	0.337
hs-CRP (mg/L)	**1.47 (0.50, 3.30)**	**1.70 (0.68, 3.28)**	**4.75 (1.10, 9.10)**	**11.012**	**0.004**
IL-2 (pg/ml)	3.11 (2.73, 3.36)	2.84 (2.59, 3.42)	3.26 (2.82, 4.20)	3.543	0.170
IL-4 (pg/ml)	2.21 (1.86, 2.51)	1.95 (1.73, 2.28)	1.97 (1.78, 2.21)	4.277	0.118
IL-6 (pg/ml)	**5.19 (4.29, 8.14)**	**6.81 (5.64, 8.83)**	**11.93 (6.70, 16.67)**	**22.672**	**<0.001**
IL-10 (pg/ml)	3.96 (3.60, 4.42)	3.68 (3.37, 4.96)	3.89 (3.42, 4.85)	0.547	0.761
Fibrinogen (g/L)	2.71 ± 1.27	2.84 ± 1.23	2.90 ± 1.15	0.276	0.759
Average heart rate (beats/min)	68.21 ± 10.07	67.74 ± 8.61	69.30 ± 6.84	0.371	0.690
Total power (ms^2^)	1814.10(1092.48, 2560.40)	1934.60(1202.40, 2692.90)	1754.00(1294.80, 2590.50)	0.151	0.927
Normalized LF power norm (nu)	63.23 (48.14, 70.57)	67.20 (55.02, 74.54)	63.33 (55.56, 73.92)	1.874	0.392
Normalized HF power norm (nu)	26.60 (15.18, 36.73)	27.46 (20.58, 35.30)	29.84 (19.28, 38.24)	1.629	0.443
LF (ms^2^)	242.20 (152.40, 422.80)	286.00 (165.40, 456.20)	359.40 (227.50, 725.50)	3.816	0.167
HF (ms^2^)	119.80 (51.10, 185.10)	95.10 (66.70, 189.70)	165.00 (89.60, 256.00)	4.580	0.101
LF/HF	2.37 (1.43, 3.53)	2.57 (1.67, 4.07)	2.49 (1.57, 3.83)	0.741	0.690
**Vessel analysis**					
cQFR of target vessel	**0.78 ± 0.25**	**0.67 ± 0.20**	**0.62 ± 0.22**	**6.183**	**0.003**
Three-vessel cQFR	**2.69 ± 0.34**	**2.49 ± 0.35**	**2.22 ± 0.44**	**16.563**	**<0.001**
Target vascular location				6.539	0.162
LAD	27 (62.8)	18 (41.9)	26 (60.5)		
LCX	7 (16.3)	11 (25.6)	11 (25.6)		
RCA	9 (20.9)	14 (32.6)	6 (14.0)		

*The meaning of the bold values are a two-sided P <0.05 was considered statistically significant*.

**Table 4:** Factors associated with the severity of coronary lesions as detected by Gensini Score.

**Independent variables**	**B**	**SE**	**WALS**	***P***	**OR**	**95%**	
						**Lower limit**	**Upper limit**
Female	−0.150	0.459	0.107	0.744	0.861	0.350	2.117
Current smoking	0.345	0.425	0.657	0.418	1.412	0.613	3.248
HDL-c	**−1.685**	**0.681**	**6.132**	**0.013**	**0.185**	**0.049**	**0.704**
hs-CRP	**0.065**	**0.033**	**3.974**	**0.046**	**1.067**	**1.001**	**1.138**
IL-6	−0.008	0.008	1.116	0.291	0.992	0.977	1.007

*The meaning of the bold values are a two-sided P < 0.05 was considered statistically significant*.

### Linear Regression Model for 3V-cQFR

**Figure 3:**
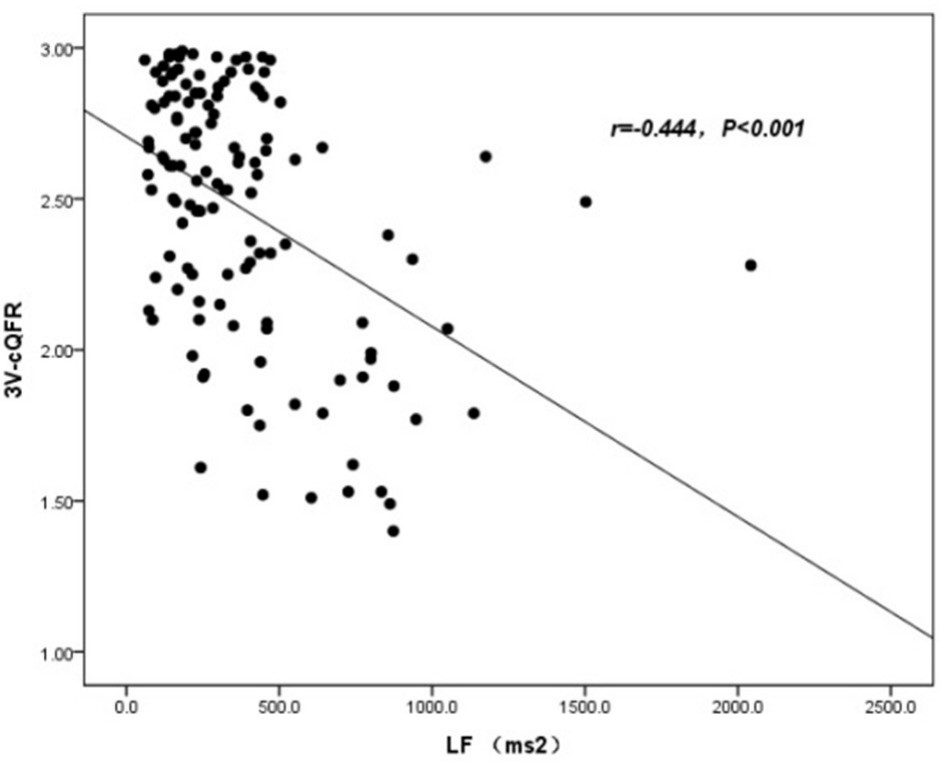
Association analysis between 3V-cQFR and LF.

**Figure 4:**
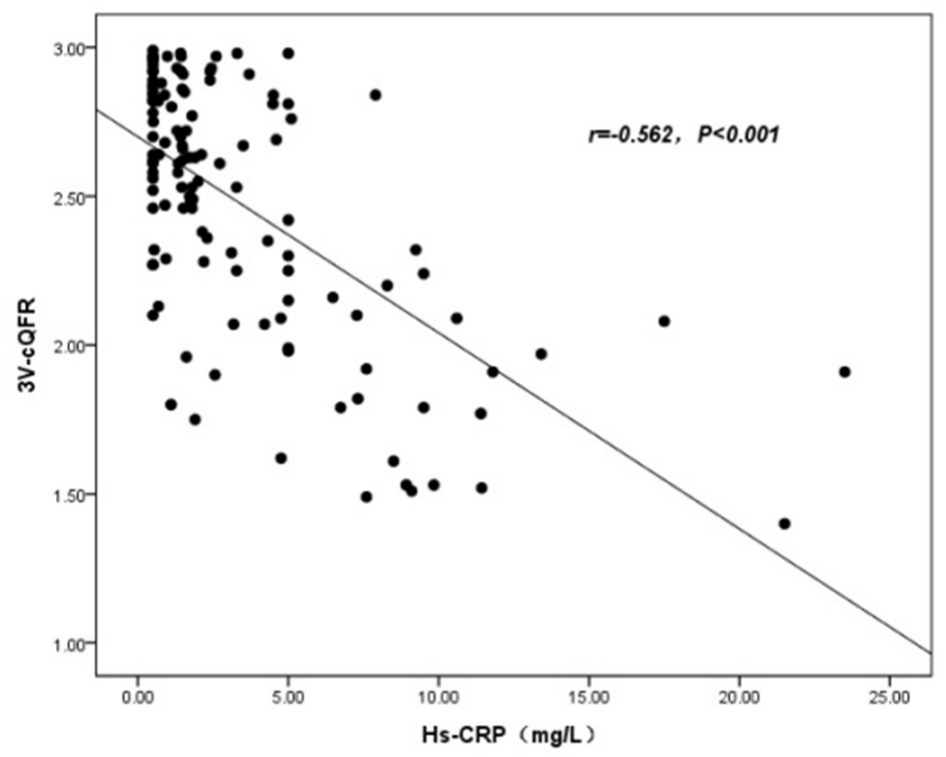
Association analysis between 3V-cQFR and hs-CRP.

The baseline features of patients based on 3V-cQFR are shown in Table 5. Those with lower 3V-cQFR were mostly male, current smoker, with a family history of CAD, lower HDL-C, and higher hs-CRP, fibrinogen, and LF (all *P* < 0.05; Table 5). Multivariate linear regression analysis showed that a family history of CAD, hs-CRP, and LF were all independently and inversely associated with 3V-cQFR (all *P* < 0.05; Table 5). LF (*r* = 0.444, *P* < 0.001; Figure 3) and hs-CRP (*r* = 0.562, *P* < 0.001; Figure 4).

**Table 5:** Patient characteristics according to baseline 3V-cQFR.

	**Univariate**			**Multivariate**		
	**B**	**T**	***p***	**B**	**T**	***p***
Female (%)	**0.286**	**3.851**	**<0.001**	0.044	0.472	0.638
Age (years)	0.004	1.052	0.295			
Hypertension (%)	−0.107	1.347	0.181			
Diabetes mellitus (%)	−0.114	1.404	0.163			
Current smoking (%)	**−0.148**	**1.995**	**0.048**	−0.002	0.018	0.985
Family history of CAD (%)	**−0.280**	**2.211**	**0.029**	**−0.276**	**2.536**	**0.012**
BMI (kg/m^2^)	−0.015	1.570	0.119			
TG (mmol/l)	0.006	0.286	0.775			
TC (mmol/l)	0.003	0.111	0.912			
HDL-c (mmol/l)	**0.369**	**3.168**	**0.002**	0.157	1.457	0.148
LDL-c (mmol/l)	−0.051	1.541	0.126			
Lp(a) (g/L)	0.001	1.640	0.103			
hs-CRP (mg/L)	**−0.005**	**2.718**	**0.007**	**−0.005**	**2.621**	**0.010**
IL-2 (pg/ml)	0.011	0.394	0.695			
IL-4 (pg/ml)	0.042	1.286	0.203			
IL-6 (pg/ml)	0.001	0.423	0.673			
IL-10 (pg/ml)	−0.004	−0.187	0.852			
Fibrinogen (g/L)	**−0.097**	**3.233**	**0.002**	−0.049	1.612	0.110
Average heart rate (beats/min)	−0.001	0.103	0.918			
Total power (ms^2^)	0.001	0.070	0.944			
LF (ms^2^)	**−0.001**	**5.705**	**<0.001**	**−0.001**	**3.128**	**0.002**
HF (ms^2^)	−0.001	1.527	0.129			
LF/HF	−0.09	1.137	0.258			

*The meaning of the bold values are a two-sided P < 0.05 was considered statistically significant*.

### Evaluation of Discrimination and Reclassification Abilities of the Predictive Models for the Functional Severity of Coronary Stenosis of Target Vessel Using cQFR

**Figure 5:**
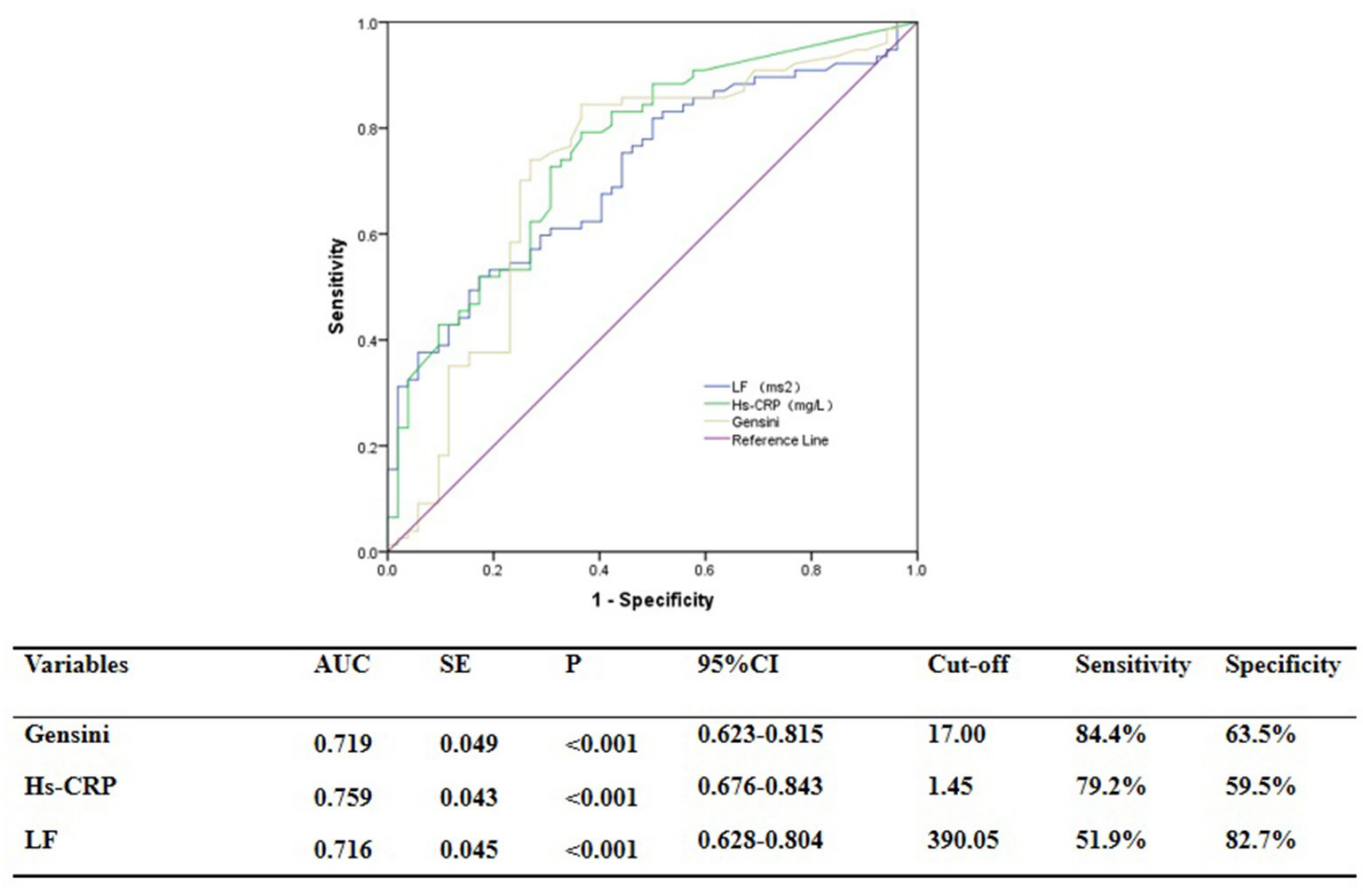
ROC analysis for the predictive efficacy of variables for functional ischemia of target vessel as detected by cQFR.

**Figure 6:**
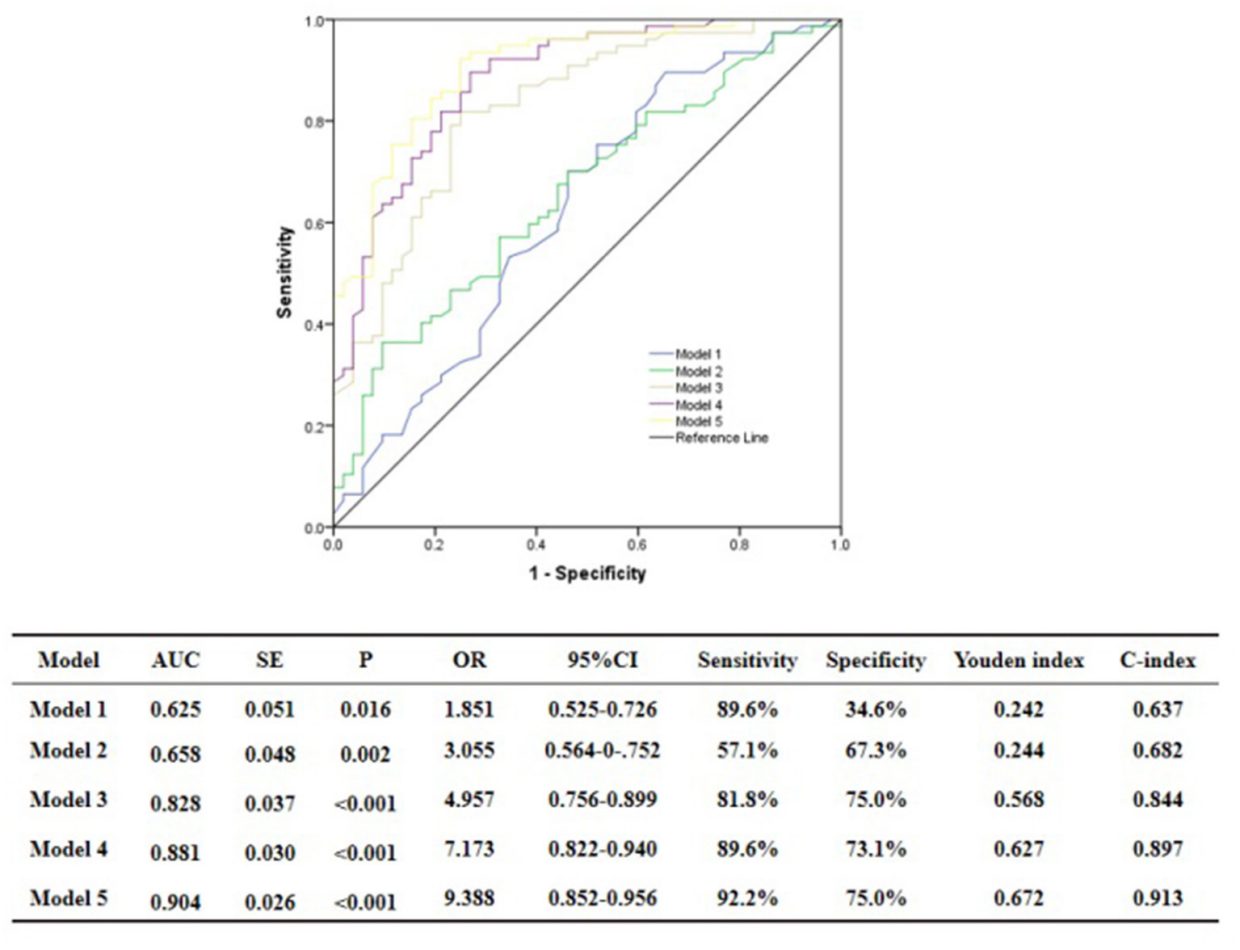
Comparison of discrimination and reclassification abilities of predictive models for functional ischemia of target vessel as detected by cQFR. Model 1, Age + Sex; Model 2, Model 1 + Hypertension + Diabetes mellitus + Current smoking + Current drinking + BMI > 28 Kg/m^2^; Model 3, Model 2 + Genisi Score > 17; Model 4, Model 3 + hs-CRP > 1.45 mg/L; Model 5, Model 4 + LF > 390.5 ms^2^.

ROC curve analysis showed that GS was predictive of the functional severity of coronary stenosis of target vessel as detected by cQFR, and a cut-off value of GS = 17.00 conferred a sensitivity of 84.4% and a specificity of 63.5%. The area under the ROC (AUC) was 0.719 for GS, suggesting good validity (*P* < 0.001; 95% confidence interval (CI): 0.623–0.815). Hs-CRP was predictive of the functional severity of coronary artery stenosis of target vessel as detected by cQFR, and a cut-off value of hs-CRP = 1.45 conferred a sensitivity of 79.2% and a specificity of 59.5%. The AUC for hs-CRP was 0.759, indicating good validity (*P* < 0.001; 95% CI: 0.676–0.843). LF was predictive of the functional severity of coronary artery stenosis of target vessel as detected by cQFR, and a cut-off value of LF = 390.05 conferred a sensitivity of 51.9% and a specificity of 82.7%. The AUC for LF was 0.716, indicating good validity (*P* < 0.001; 95% CI: 0.628–0.804) (Figure 5). In the multivariable analysis model, GS, hs-CRP, and LF increased the discriminatory indices when added to clinical risk factors (Figure 6). In model 3, the increase of GS also significantly increased the ability to accurately predict the functional severity of coronary artery stenosis of target vessel compared with model 2 using conventional cardiovascular risk factors (AUC: 0.828; C-index: 0.844; Youden index: 0.568; sensitivity: 81.8%; specificity: 75.0%; *P* < 0.001). For the predictability of the functional severity of coronary stenosis of target vessel, the positive Youden index of the combined hs-CRP increased in model 4 (AUC: 0.881; C-index: 0.897; Youden index: 0.672; sensitivity: 89.6%; specificity: 73.1%; *P* < 0.001) (Figure 6). Adding LF > 390.05 into model 5 further increased the discriminatory and reclassification indices for the occurrence of the functional severity of coronary stenosis of target vessel (AUC: 0.904; C-index: 0.913; Youden index: 0.672; sensitivity: 92.2%; specificity: 75.0%; CI: 0.852–0.956; *P* < 0.001) (Figure 6).

## Discussion

For patients with new-onset UAP, we found that (1) plasma hs-CRP and LF were independently associated with the functional severity of coronary artery stenosis of target vessel, as well as the total atherosclerotic burden, as determined by cQFR, and LF was positively correlated with hs-CRP; (2) higher plasma hs-CRP at baseline was independently correlated with the severity of coronary artery lesion as evaluated by GS, while LF was not associated with GS; and (3) integration of the increase of LF into hs-CRP and GS significantly increased the discriminatory ability and accuracy in the prediction of the functional severity of coronary artery stenosis of target vessel.

### HRV and cQFR Measurement

Although coronary angiogram is the gold standard for diagnosing CAD, the degree of anatomical stenosis of the coronary artery does not always reflect the severity of coronary myocardial ischemia (3, 4, 26, 27). cQFR is a new diagnostic method for the functional evaluation of coronary artery stenosis that can precisely predict the clinical outcome of patients with ACS (17, 26, 27). Notably, assessment of the physiological functions of ‘non-culprit’ vessels could help predict future cardiovascular adverse events in “vulnerable” patients even if they are symptom-free (6, 28, 29). Previous studies have provided new insights into the total “anatomical” atherosclerotic burden (6), and we believe it is reasonable to suggest that 3V-cQFR can be used as an index for total “physiological” atherosclerotic burden and identification of “vulnerable” patients. Moreover, previous studies suggested that cardiac sympathetic nerve modulation reflected by HRV could accelerate coronary atherosclerosis via its pro-inflammatory and prothrombotic effects (13, 30). Thus, HRV in patients with no known history of CAD may serve as a biomarker for myocardial ischemia (31). Our study further showed that LF and LFn, reflecting cardiac sympathetic nerve modulation, were associated with functional ischemia, and LF was an independent risk factor for evaluating the functional stenosis of the target vessels and total atherosclerotic burden. Reports have shown that non-cardiovascular pathological conditions, such as respiratory, neurologic, and renal disease, can affect HRV (23, 32). In addition, the current approaches applied to analyze the long-term HRV, such as the 24-h heart rate, are based on the stationary assumption (33–37). However, these methods are still widely used for non-stationary time series, including the 24–48-h long-term heart rate (23, 38). In our study, the average 5-minute short term HRV was used to analyze normal sinus rhythm, as this method has been used an indicator of the combined effect of cardiac regulation by baroreflex, neurohormones, physical and mental activities, and circadian rhythm of daily living. Compared with 24-h (long-term) analysis, we used the relatively accurate method to obtain indexes in the frequency-domain parameters (23, 38). Moreover, rigorous inclusion and exclusion criteria were adopted in our study to reduce the influence of HRV (23).

Since HRV is closely correlated with CAD and cardiogenic death, and past CAD may affect HRV that ultimately affects the accuracy of our conclusions, patients with a history of CAD were excluded from our study. Moreover, to the best of our knowledge, the correlation between HRV and new-onset UAP has not been established. Previous studies have shown correlations between HRV and the development and progression of atherosclerosis (12), and low HRV has been identified as an independent predictor for cardiovascular mortality and sudden cardiac death (21, 39). Our results are inconsistent with previous studies showing that lower LF was associated with the severity of coronary lesion in patients who underwent angiography (12). Of note, coronary arteriography was intended to evaluate the severity of coronary artery lesions but not the functional severity of coronary artery stenosis. Interestingly, using HRV analysis combined with the HeartTrends DyDx algorithm, another study showed that HRV was correlated with myocardial ischemia through exercise stress echocardiography and exercise myocardial perfusion imaging (31). However, all patients who did not undergo coronary angiography assessing the coronary artery stenosis were included in that study. Likewise, patients with myocardial ischemia were shown to have heightened sympathetic modulation relative to parasympathetic modulation, which suggested that augmented sympathetic modulation is the burden of ischemia (40). Moreover, accelerated heart rate is an early marker of myocardial ischemia (41, 42). Since QFR yields a consistent evaluation of FFR that can be used to evaluate functional ischemia of target vessel and total atherosclerotic burden using the 3V-cQFR measurement (6, 17, 26), our study provides a more accurate association of these factors. Contrary to previous studies that LF was inversely correlated with the severity of coronary stenosis (12, 21, 39), we found that LF was directly correlated with the functional severity of coronary artery stenosis. Therefore, we speculate that, in the early stages of myocardial ischemia, there is elevated sympathetic nerve tone in patients with new-onset UAP, leading to an increase in LF, a potential early marker of myocardial ischemia. However, autonomic dysfunction may occur as the CAD progresses, which may lead to a concomitant reduction in LF.

### Inflammation and cQFR Measurement

Currently, the “inflammatory hypothesis of atherothrombosis” is the popular theory of atherosclerosis pathogenesis (43, 44). Studies have demonstrated that an increase in hs-CRP and IL-6 may confer similar risk as conventional cardiovascular risk factors for the incidence and prognostication of myocardial infarction and atherosclerosis (19, 45–47). Mounting literature acknowledges the importance of evaluating the association between biomarkers of inflammation and CAD risk, which predicts the severity of coronary stenosis and clinical outcomes. In addition, a previous study showed that inflammation might be the triggering mechanism in most, but not all, patients with ACS, suggesting that there are individual differences in the inflammatory response in patients with ACS (48). We used QFR to evaluate not only functional ischemia of target vessel but also the total atherosclerotic burden, including the functional evidence of the non-culprit vessels. We uncovered the pathophysiological basis underlying the association between biomarkers of inflammation and coronary artery events by showing that hs-CRP is associated with the functional severity of coronary artery stenosis. (32). Furthermore, hs-CRP independently predicted the severity of coronary artery lesions by GS, as evidenced by multiple stepwise logistics regression analysis in patients with new-onset UAP. Therefore, our data support that an increased hs-CRP may be an early marker of myocardial ischemia and is directly correlated with the severity of cardiovascular disease.

### Autonomic Nervous System and Inflammation

**Figure 7:**
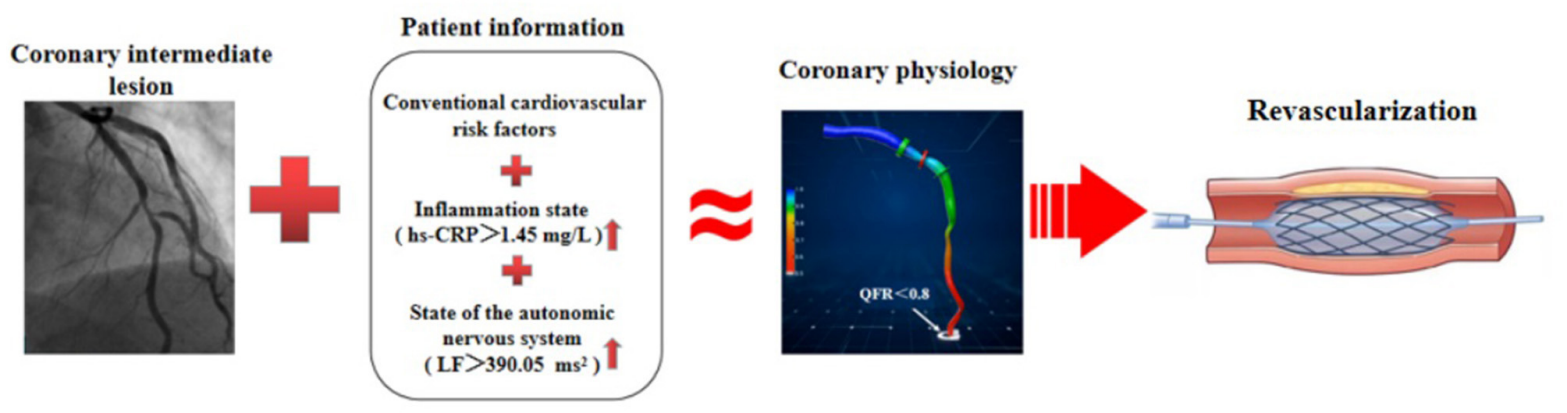
Schematic diagram for evidence to diagnose functional ischemia. In the absence of QFR analysis, model-based risk assessment including LF, hs-CRP, GS, and traditional risk factors, can predict functional ischemia in patients with coronary intermediate lesions and inform whether or not to schedule PCI.

Emerging evidence demonstrates that the autonomic nervous system modulates inflammatory responses (32). Recently, observational and translational studies found that in response to environmental noise, the sympathetic nervous system becomes activated, which activates several pro-inflammatory pathways, leading to vascular inflammation and endothelial injury that accelerate lipid deposition and recruitment of more inflammatory cells into blood vessels, and thus the development of CAD (14). Our results showed that LF was directly associated with hs-CRP, which is consistent with previous studies showing that multiple mechanisms, especially inflammation, were responsible for the potential role of sympathetic excitation in accelerating coronary artery atherosclerosis (14–16). Importantly, given that model-based risk assessment including LF, hs-CRP, GS, and traditional risk factors can predict functional ischemia in patients with new-onset UAP, non-urgent stent implantations and other dispensable revascularization procedures can be deferred, even in the absence of QFR analysis (Figure 7). These findings indicate that an imbalance in cardiac autonomic control is correlated with increased systemic inflammation in patients with new-onset UAP. Our data also confirmed that excessive sympathetic activation promotes the proliferation, differentiation, and mobilization of bone marrow hematopoietic stem cells and progenitor cells, and increases the number of pro-inflammatory monocytes in circulation, which has been shown to accelerate atherosclerosis (14–16, 49). More studies are required to validate our findings and confirm the importance of monitoring the autonomic nervous system and systemic inflammation in patients with UAP.

### Study Limitations

There are some limitations in our study. First, this was a retrospective observational study with a relatively small sample size from a single center. Thus, we could not prevent selection bias. The findings should be validated in prospective studies with larger samples from multiple centers. Second, the majority of cQFR computation required manual correction for tracing, and the present study did not use FFR as a gold standard for the control group, which could reduce the generalizability of study. Third, since almost one third of selected patients were diabetic and observational data showed discordance between cQFR and FFR in patients with diabetes, the functional ischemia in patients with UAP needs to be further verified using the gold-standard FFR procedure. Fourth, our study did not use optical coherence tomography or intravascular ultrasound to analyze plaque burden or characteristics, and 3V-cQFR could not represent the total plaque burden because diseased side branches and ostial lesions were not analyzed. Finaly, a lack of longitudinal follow-up data prohibited assessment of the clinical impact of QFR analysis on future events.

## Conclusions

In patients with UAP, higher LF and hs-CRP were independently associated with increased risk of functional ischemia and total atherosclerotic burden, as evaluated by cQFR. An imbalance of cardiac autonomic regulation was related to accelerated systemic inflammation in patients with new-onset UAP. Measurement of HRV and hs-CRP may add valuable information for the early diagnosis of functional ischemia and serve as a reliable parameter to decide whether or not to schedule percutaneous transluminal coronary intervention for patients with new-onset UAP, even in the absence of QFR analysis.

## Data Availability Statement

The datasets used and/or analyzed during this study are available from the corresponding author on reasonable request. Requests to access these datasets should be directed to Hong Jiang, hong-jiang@whu.edu.cn.

## Ethics Statement

Because this was a retrospective observational study, the Renmin Hospital of Wuhan University Ethics Committee granted an exemption from requiring ethics approval and informed consent from eligible patients was waived.

## Author Contributions

LY and HJ: substantial contributions to conception and design, data acquisition, or data analysis and interpretation. JW, WL, HuC, CL, MW, HuaC, HZ, ZL, SZ, ZY, SD, QD, and JS: drafting the article or critically revising it for important intellectual content. JW, WL, HuaC, CL, and MW: final approval of the version to be published and agreement to be accountable for all aspects of the work in ensuring that questions related to the accuracy or integrity of the work are appropriately investigated and resolved. All authors contributed to the article and approved the submitted version.

## Conflict of Interest

The authors declare that the research was conducted in the absence of any commercial or financial relationships that could be construed as a potential conflict of interest.

## Publisher's Note

All claims expressed in this article are solely those of the authors and do not necessarily represent those of their affiliated organizations, or those of the publisher, the editors and the reviewers. Any product that may be evaluated in this article, or claim that may be made by its manufacturer, is not guaranteed or endorsed by the publisher.
